# The Association Between Prenatal Anxiety and Depression and Autism Spectrum Disorder With Gastrointestinal Symptoms in Children: Case-Control Study

**DOI:** 10.2196/90446

**Published:** 2026-10-28

**Authors:** Boli Cheng, Enyu Wan, Yuanxia He, Si Wang, Yun He, Xiaojiang Lyu

**Affiliations:** 1 Department of Pediatrics, Affiliated Hospital of North Sichuan Medical College, Nanchong, Sichuan, 637000, China, 86 0817 2262406, 86 0817-2262406; 2 Department of Clinical Medicine, North Sichuan Medical College, Nanchong, Sichuan, China

**Keywords:** autism spectrum disorder, gastrointestinal symptoms, comorbidity, risk factor, prenatal

## Abstract

**Background:**

Studies have indicated that maternal anxiety and depression during pregnancy can contribute to neurodevelopmental disorders and gut flora dysbiosis in children. However, it is unclear whether these factors increase the risk of autism spectrum disorder (ASD) with gastrointestinal (GI) symptoms in children.

**Objective:**

This study aimed to analyze the relationship between maternal anxiety and depression during pregnancy and ASD with GI symptoms.

**Methods:**

A total of 470 children with ASD and 486 children with typical development were enrolled in the study. The caregivers of all enrolled children completed questionnaires, including information on maternal mental health and GI symptoms.

**Results:**

The percentage of maternal prenatal anxiety or depression was significantly higher in the ASD group than in the typical development group (*P*<.001). Children whose mothers experienced anxiety or depression during pregnancy had substantially higher risk of developing ASD than those whose mothers did not experience these conditions (unadjusted odds ratio [OR] 4.274, 95% CI 2.799-6.526; adjusted OR 3.897, 95% CI 2.481-6.119). For children with ASD, those whose mothers had prenatal anxiety or depression had a significantly higher risk of GI symptoms than those whose mothers did not have these conditions (unadjusted OR 1.883, 95% CI 1.209-2.934; adjusted OR 1.943, 95% CI 1.236-3.054).

**Conclusions:**

Mothers who experience anxiety or depression during pregnancy may have a higher likelihood of their children developing ASD with GI symptoms.

## Introduction

Autism spectrum disorder (ASD) is a neurodevelopmental disorder that has an early onset and significantly affects children’s health. The common core symptoms of ASD are impaired social skills and communication and restricted or repetitive behaviors [1]. The etiology and pathogenesis of ASD are still not well understood. It is currently accepted that both genetic and environmental factors play key roles in the development of ASD [1]. Environmental conditions during early life, such as exposures during maternal pregnancy, may impact the neurodevelopment of children and increase the risk of ASD.

The manifestations of ASD are highly heterogeneous. In addition to the core symptoms, there are often comorbidities such as sleep disorders, attention-deficit/hyperactivity disorder, and gastrointestinal (GI) symptoms [2]. The etiology of ASD is also complex and cannot be explained by a single factor. Thus, certain causes may contribute to both core symptoms and comorbidities of ASD. GI symptoms are a common comorbidity of ASD, and quite a number of studies have shown that the prevalence of GI symptoms in children with ASD is much higher than in children with typical development [3]. As such, some factors may affect both early neurodevelopment and GI function, potentially leading to the development of ASD and GI symptoms.

The prenatal period is crucial for the neurodevelopment of children, and various environmental factors can greatly influence this development [4,5]. One important environmental factor is the mother’s mental health status during pregnancy. Due to hormonal changes and other factors, some pregnant women who have no history of mental illness may develop psychological disorders, and thus, there is a relatively high prevalence of anxiety and depression during pregnancy [6,7]. Studies have demonstrated that maternal prenatal anxiety and depression can affect the neurodevelopment of children and may be associated with neurodevelopmental disorders such as attention-deficit/hyperactivity disorder and cognitive disorders [8-10]. In addition, some studies have reported associations between maternal prenatal anxiety and depression and ASD in children. For instance, a large cohort study found that prenatal depression was associated with increased ASD-related traits in children [11]. Similarly, a meta-analysis reported an increased risk of ASD in children whose mothers experienced depressive disorders [12]. Another recent systematic review and meta-analysis demonstrated that maternal prenatal mood and anxiety disorders were associated with a significantly increased risk of ASD in children [13]. Pregnancy is also a critical period for the development of the enteric nervous system (ENS), and maternal anxiety and depression during this time have been found to alter the intestinal microbiota of children [14]. We speculate that maternal prenatal anxiety and depression may be linked to ASD and comorbid GI symptoms in children by simultaneously affecting the development of both the central nervous system and the ENS or by influencing children’s gut microbiota and thereby affecting the development of both the brain and the gut via the gut-brain axis. Nevertheless, the relationship between prenatal anxiety and depression and GI symptoms in children is unclear, and the relationship between maternal anxiety and depression during pregnancy and ASD with GI symptoms has not been reported. Maternal anxiety and depression during pregnancy are mostly mild to moderate [15], but these symptoms may not be fully recognized and treated, partly because pharmacological interventions during pregnancy require particular caution and nonpharmacological interventions are not always readily available [16]. The purpose of this study was to analyze the relationship between mild and moderate maternal prenatal anxiety or depression and ASD in children, as well as the potential link between these conditions and ASD with GI symptoms.

## Methods

### Participants

A total of 470 children with ASD aged 2 to 7 years who visited the child psychobehavioral clinic of our hospital from July 2022 to June 2024 were recruited. The child psychobehavioral clinic is a specialized outpatient clinic within an affiliated hospital of a medical college primarily responsible for the diagnosis and treatment of developmental and behavioral disorders. Recruitment was conducted systematically: all children who visited the clinic with suspected or previously diagnosed ASD were screened for eligibility by the attending clinician. Then, a research coordinator contacted eligible families and explained the study purpose, procedures, and confidentiality protections. Families who agreed to participate were then scheduled for a comprehensive evaluation. The diagnosis of ASD was confirmed by 2 developmental-behavioral pediatricians based on the diagnostic criteria defined in the *Diagnostic and Statistical Manual of Mental Disorders*, *Fifth Edition* [17], after a series of structured interviews and in combination with the results of the Childhood Autism Rating Scale (CARS) assessment. The CARS is a widely used standardized assessment scale for ASD symptoms, and individuals with a total CARS score of 30 or higher are considered to have ASD characteristics [18]. The exclusion criteria were history of developmental disabilities other than ASD or neurological disorders (children with ASD and co-occurring developmental disabilities were not excluded), severe physical or sensory impairment, no medical records of maternal mental health during pregnancy, history of maternal prepregnancy mental illness or paternal mental illness, physician-advised antidepressants or use of other medications (eg, antipsychotics, mood stabilizers, or anxiolytics) or substances (eg, alcohol, tobacco, or illicit drugs) during maternal pregnancy, and unwillingness to participate. Of the 522 initially screened children with ASD, 52 (10%) were excluded: 29 (5.6%) due to meeting the exclusion criteria and 23 (4.4%) due to parental refusal, resulting in a final sample size of 470 (90%). There were no missing data for the primary variables in the ASD group.

During the same period, 486 children with typical development were recruited from the child health clinic of the same hospital. They were frequency matched to the ASD group by sex and age. This clinic provides regular monitoring and guidance on the growth and development of children. Recruitment was conducted systematically: researchers approached families of children aged 2 to 7 years who attended the clinic for routine health checkups. After obtaining verbal consent, the researchers screened the children to ensure they met the inclusion criteria. Families who met the criteria were then invited to participate. The exclusion criteria were history of developmental disabilities or neurological disorders, severe physical or sensory impairment, no medical records of maternal mental health during pregnancy, history of maternal prepregnancy mental illness or paternal mental illness, physician-advised antidepressants or use of other medications (eg, antipsychotics, mood stabilizers, or anxiolytics) or substances (eg, alcohol, tobacco, or illicit drugs) during maternal pregnancy, and unwillingness to participate. Of the 532 children with typical development initially screened, 46 (8.6%) were excluded: 17 (3.2%) due to meeting the exclusion criteria and 29 (5.5%) due to parental refusal, resulting in a final sample size of 486 (91.4%). All enrolled participants with typical development completed the assessments, and there were no missing data for core variables in this group.

### Ethical Considerations

The study protocol was approved by the ethics committee of the Affiliated Hospital of North Sichuan Medical College (2022ER268-1). Informed consent was obtained from the parents of all participants.

### Questionnaires

Caregivers of all enrolled children, both those with ASD and those with typical development, completed questionnaires under the guidance of trained investigators, including questionnaires on general demographic information, maternal mental health during pregnancy, and GI symptoms. Prenatal anxiety or depression was determined based on 2 sources: scores on the Edinburgh Postnatal Depression Scale (cutoff of 10 or higher for depression) [19] and scores on the Self-Rating Anxiety Scale (cutoff of 50 or higher for anxiety) [20,21] completed during pregnancy, as well as medical records indicating clinical diagnoses of anxiety or depression by pregnancy care physicians. Only mothers who scored above the cutoff on the relevant scale and had a documented medical diagnosis were classified as having prenatal anxiety or depression. The GI symptom questions related to constipation, diarrhea, abdominal pain, flatulence, and nausea and vomiting were all based on the Questionnaire of Pediatric Gastrointestinal Symptoms–Rome IV (QPGS-IV), a validated tool for diagnosing functional GI disorders in infants and children [22]. The QPGS-IV defines a “GI symptom” as meeting specific frequency and duration thresholds (eg, diarrhea requires ≥4 loose stools per day for at least 1 month). As stool odor is not clearly defined in the QPGS-IV, it was defined as abnormal odor of the stool, such as rotten egg smell in this study [23]. A child was classified as screening positive for GI symptoms if at least 1 of the 6 GI symptoms was positive.

### Covariates

The covariates we considered were age, sex, maternal educational level, paternal educational level, age of the mother at conception, mode of delivery, preterm birth, place of residence, and annual household income. Among these, maternal educational level, paternal educational level, mode of delivery, place of residence, and annual household income were significantly different between the ASD and typical development groups. Age, sex, and covariates that were significantly different between the ASD and typical development groups were all included in the logistic regression model.

### Statistical Analyses

The data analysis was conducted using the SPSS statistical software (version 25; IBM Corp). Before analysis, the distribution of each dataset was tested for normality using the Kolmogorov-Smirnov goodness-of-fit test. The nonnormally distributed continuous variables were described using medians and IQRs, and differences between groups were assessed using the Mann-Whitney *U* test. Categorical variables were described as frequencies and percentages, and differences between groups were assessed using the chi-square test. Binary logistic regression was used to evaluate the relationship between prenatal anxiety or depression and the risk of ASD in children, as well as the link between prenatal anxiety or depression and the risk of GI symptoms in children with ASD.

## Results

### Characteristics of the Participants in the Typical Development and ASD Groups

The present study included 470 children with ASD (388 male and 82 female) and 486 children with typical development (388 male and 98 female; Figure 1). The median age of the ASD group was 4.0 (IQR 3.2-4.9) years, and that of the typical development group was 4.3 (IQR 3.1-5.3) years. There were no significant differences in sex ratio or age distribution between the 2 groups. As shown in Table 1, there were significant differences between the 2 groups in terms of both the maternal and paternal educational levels (*P*<.001), with increased proportions of both mothers and fathers with lower educational levels and decreased proportions of those with higher educational levels in the ASD group compared to the typical development group. There were no significant differences in the age of mothers at conception (*P*=.70) or the proportion of preterm births (*P*=.40) between the 2 groups. However, the proportion of vaginal delivery was significantly lower, and the proportion that lived in urban areas was also significantly lower in the ASD group than in the typical development group (*P*<.01). The annual household income was significantly different between the 2 groups (*P*<.001). In the ASD group, the proportion of those with an income below CN¥ 50,000 (CN¥ 1=US $0.15 as of October 15, 2026) was higher, while that of those with an income above CN¥ 100,000 was significantly lower.

**Figure 1.**
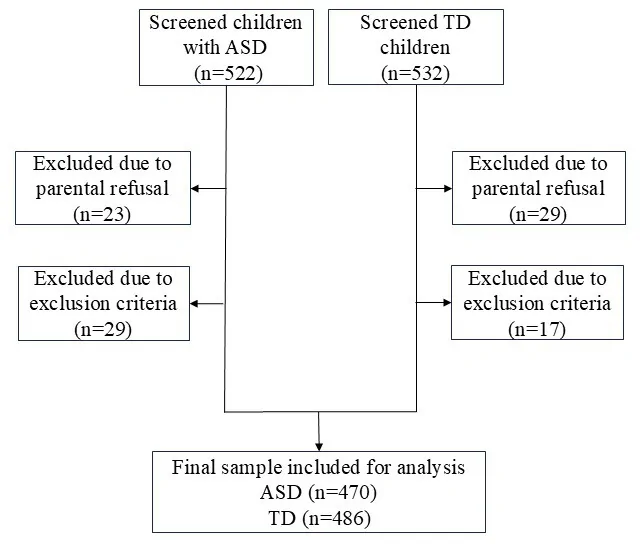
Flowchart of participant recruitment. ASD: autism spectrum disorder; TD: typical development.

**Table 1.** Characteristics of the participants in the typical development (TD) and autism spectrum disorder (ASD) groups.

Variables	TD (n=486)	ASD (n=470)	*z* or chi-square (*df*)	*P* value
Age (y), median (IQR)	4.3 (3.1-5.3)	4.0 (3.2-4.9)	–1.507^a^	.13
Sex, n (%)			1.2 (1)^b^	.28
Male	388 (79.8)	388 (82.6)		
Female	98 (20.2)	82 (17.4)		
Maternal educational level, n (%)			68.8 (2)^b^	<.001
Primary school or lower	1 (0.2)	11 (2.3)		
Secondary school	109 (22.4)	213 (45.3)		
College or higher	376 (77.4)	246 (52.3)		
Paternal educational level, n (%)			46.3 (2)^b^	<.001
Primary school or lower	1 (0.2)	12 (2.6)		
Secondary school	113 (23.3)	191 (40.6)		
College or higher	372 (76.5)	267 (56.8)		
Age of mother at conception (y), n (%)			0.2 (1)^b^	.70
<35	443 (91.2)	425 (90.4)		
≥35	43 (8.8)	45 (9.6)		
Mode of delivery, n (%)			9.2 (1)^b^	.002
Vaginal	270 (55.6)	215 (45.7)		
Cesarean	216 (44.4)	255 (54.3)		
Preterm birth, n (%)			0.7 (1)^b^	.40
Yes	59 (12.1)	49 (10.4)		
No	427 (87.9)	421 (89.6)		
Place of residence, n (%)			44.2 (1)^b^	<.001
Urban	448 (92.2)	360 (76.6)		
Rural	38 (7.8)	110 (23.4)		
Annual household income (CN¥; CN¥ 1=US $0.15 as of October 15, 2026), n (%)			121.7 (2)^b^	<.001
<50,000	121 (24.9)	236 (50.2)		
50,000-100,000	110 (22.6)	145 (30.9)		
>100,000	255 (52.5)	89 (18.9)		
Prenatal anxiety or depression, n (%)			50.9 (1)^b^	<.001
Yes	31 (6.4)	106 (22.6)		
No	455 (93.6)	364 (77.4)		
GI^c^ symptoms, n (%)			56.7 (1)^b^	<.001
Yes	129 (26.5)	236 (50.2)		
No	357 (73.5)	234 (49.8)		

a *z* statistic.

b chi-square statistic.

c GI: gastrointestinal.

In the ASD group, 22.6% (106/470) of the individuals had maternal prenatal anxiety or depression, while only 6.4% (31/486) in the typical development group did. The percentage of maternal prenatal anxiety or depression was significantly higher in the ASD group than in the typical development group (*P*<.001).

In addition, 50.2% (236/470) of the children in the ASD group had GI symptoms compared with 26.5% (129/486) in the typical development group. The GI symptoms were significantly higher in the ASD group than in the typical development group (*P*<.001).

### Associations Between Maternal Prenatal Anxiety or Depression and the Risk of ASD in Children

To examine the potential link between maternal prenatal anxiety or depression and ASD in children, we conducted logistic regression analysis. The results, shown in Table 2, indicate that in univariate logistic regression, prenatal anxiety or depression (odds ratio [OR] 4.274, 95% CI 2.799-6.526), lower maternal educational level (secondary school: OR 2.987, 95% CI 2.255-3.957; primary school or lower: OR 16.813, 95% CI 2.157-131.049), lower paternal educational level (secondary school: OR 2.355, 95% CI 1.779-3.118; primary school or lower: OR 16.719, 95% CI 2.161-129.360), nonvaginal delivery (OR 1.483, 95% CI 1.149-1.913), and nonurban residence (OR 3.560, 95% CI 2.400-5.281) were all associated with an increased risk of ASD, while higher household income (CN¥ 50,000-100,000: OR 0.676, 95% CI 0.485-0.941; >CN¥ 100,000: OR 0.179, 95% CI 0.129-0.248) was linked to a reduced risk of ASD. To assess independent effects, we included all significant variables along with age and sex into a multivariable logistic regression model. The results showed that maternal prenatal anxiety or depression remained a significant risk factor for ASD (adjusted OR 3.897, 95% CI 2.481-6.119). Among the other variables, maternal educational level (secondary school vs college or higher: adjusted OR 1.865, 95% CI 1.232-2.823), delivery mode (adjusted OR 1.387, 95% CI 1.042-1.845), residence (adjusted OR 2.076, 95% CI 1.326-3.252), and household income (>CN¥ 100,000 vs <CN¥ 50,000: adjusted OR 0.252, 95% CI 0.176-0.362) also remained significant but with smaller effect sizes than prenatal anxiety or depression. Paternal educational level was no longer significant (*P*>.05).

**Table 2.** Univariate and multivariate logistic regression analysis of factors potentially associated with autism spectrum disorder.

Variables	Univariate		Multivariate	
	OR^a^ (95% CI)	*P* value	OR (95% CI)	*P* value
Age (y)	0.926 (0.836-1.027)	.15	0.863 (0.767-0.970)	.01
Sex				
Female	1 (reference)	—^b^	1 (reference)	—
Male	1.195 (0.863-1.655)	.28	1.169 (0.810-1.687)	.41
Maternal educational level				
College or higher	1 (reference)	—	1 (reference)	—
Secondary school	2.987 (2.255-3.957)	<.001	1.865 (1.232-2.823)	.003
Primary school or lower	16.813 (2.157-131.049)	.007	3.921 (0.374-41.073)	.25
Paternal educational level				
College or higher	1 (reference)	—	1 (reference)	—
Secondary school	2.355 (1.779-3.118)	<.001	0.865 (0.569-1.316)	.50
Primary school or lower	16.719 (2.161-129.360)	.007	4.505 (0.437-46.415)	.21
Mode of delivery				
Vaginal	1 (reference)	—	1 (reference)	—
Cesarean	1.483 (1.149-1.913)	.002	1.387 (1.042-1.845)	.02
Place of residence				
Urban	1 (reference)	—	1 (reference)	—
Rural	3.560 (2.400-5.281)	<.001	2.076 (1.326-3.252)	.001
Annual household income (CN¥; CN¥ 1=US $0.15 as of October 15, 2026)				
<50,000	1 (reference)	—	1 (reference)	—
50,000-100,000	0.676 (0.485-0.941)	.02	0.804 (0.565-1.146)	.23
>100,000	0.179 (0.129-0.248)	<.001	0.252 (0.176-0.362)	<.001
Prenatal anxiety or depression				
No	1 (reference)	—	1 (reference)	—
Yes	4.274 (2.799-6.526)	<.001	3.897 (2.481-6.119)	<.001

a OR: odds ratio.

b Not applicable.

### Comparison of GI Symptoms and Their Association With Maternal Prenatal Anxiety or Depression in Children With ASD

We compared the GI symptoms in children with ASD whose mothers had and did not have prenatal anxiety and depression. As shown in Table 3, a total of 62.3% (66/106) of children with ASD whose mothers had prenatal anxiety or depression exhibited at least one GI symptom, while 46.7% (170/364) of children with ASD whose mothers did not have prenatal anxiety and depression exhibited GI symptoms. Our analysis revealed that children with ASD whose mothers had prenatal anxiety or depression had a significantly higher proportion of GI symptoms than those whose mothers did not have prenatal anxiety and depression (*P*<.01). Specifically, the rates of constipation and abdominal pain were significantly higher in children with ASD whose mothers had anxiety or depression during pregnancy (*P*<.01). However, there was no significant difference in the prevalence of diarrhea, nausea and vomiting, flatulence, and stool odor between the 2 groups (*P*>.05).

**Table 3.** Comparison of gastrointestinal (GI) symptoms between children with autism spectrum disorder (ASD) whose mothers had and did not have prenatal anxiety and depression.

Symptoms	ASD without prenatal anxiety or depression (n=364), n (%)	ASD with prenatal anxiety or depression (n=106), n (%)	Chi-square (*df*)	*P* value
Constipation	105 (28.8)	47 (44.3)	9.0 (1)	.003
Diarrhea	25 (6.9)	10 (9.4)	0.8 (1)	.38
Abdominal pain	43 (11.8)	25 (23.6)	9.2 (1)	.002
Nausea or vomit	24 (6.6)	9 (8.5)	0.5 (1)	.50
Flatulence	18 (4.9)	10 (9.4)	3.0 (1)	.09
Stool odor	36 (9.9)	14 (13.2)	1.0 (1)	.33
Total GI symptoms	170 (46.7)	66 (62.3)	8.0 (1)	.005

We further assessed the relationship between maternal prenatal anxiety or depression and the risk of GI symptoms in children with ASD. Our findings, as shown in Table 4, indicate that children with ASD whose mothers experienced anxiety or depression during pregnancy had a significantly higher risk of total GI symptoms than children whose mothers did not have these conditions (OR 1.883, 95% CI 1.209-2.934). This trend remained consistent even after adjusting for age, sex, maternal educational level, paternal educational level, mode of delivery, place of residence, and annual household income (adjusted OR 1.943, 95% CI 1.236-3.054). Specifically, the risk of constipation and abdominal pain was significantly higher in children with ASD whose mothers had prenatal anxiety or depression than in those whose mothers did not have these conditions (OR 1.965, 95% CI 1.259-3.067 and OR 2.304, 95% CI 1.329-3.993, respectively). These results were still evident after adjusting for covariates (adjusted OR 2.083, 95% CI 1.315-3.299 and adjusted OR 2.302, 95% CI 1.305-4.062, respectively). However, there was no significant increase in the risk of diarrhea, nausea and vomiting, flatulence, or stool odor in children with ASD whose mothers had prenatal anxiety or depression compared to those whose mothers did not have these conditions (*P*>.05).

**Table 4.** Association between maternal prenatal anxiety or depression and the risk of gastrointestinal (GI) symptoms in children with autism spectrum disorder (ASD).

GI comorbidities in ASD	Unadjusted		Adjusted^a^	
	OR^b^ (95% CI)	*P* value	OR (95% CI)	*P* value
Constipation	1.965 (1.259-3.067)	.003	2.083 (1.315-3.299)	.002
Diarrhea	1.412 (0.656-3.043)	.38	1.530 (0.692-3.385)	.29
Abdominal pain	2.304 (1.329-3.993)	.003	2.302 (1.305-4.062)	.004
Nausea and vomiting	1.314 (0.591-2.921)	.50	1.335 (0.581-3.067)	.50
Flatulence	2.002 (0.895-4.480)	.09	2.154 (0.922-5.031)	.08
Stool odor	1.386 (0.717-2.680)	.33	1.272 (0.646-2.505)	.49
Total GI symptoms	1.883 (1.209-2.934)	.005	1.943 (1.236-3.054)	.004

a Adjusted for age, sex, maternal educational level, paternal educational level, mode of delivery, place of residence, and annual household income.

b OR: odds ratio.

## Discussion

### Principal Findings

The clinical manifestation of ASD is highly heterogeneous, often with comorbidities such as GI symptoms in addition to the core symptoms [24]. Certain causes may lead to developmental abnormalities in both the brain and GI tract, resulting in ASD with GI symptoms. In this study, we investigated the impact of maternal mental health, specifically anxiety or depression during pregnancy, on the likelihood of ASD and GI symptoms in children.

In our study, we found that the prevalence of maternal prenatal anxiety or depression was 6.4% (31/486) in children with typical development and 22.6% (106/470) in children with ASD. This is a relatively high prevalence and is consistent with findings from other studies [9,25]. Our study also revealed a significant difference in the prevalence of maternal prenatal anxiety or depression between children with ASD and children with typical development, suggesting a potential link between prenatal mental health and the development of ASD in children. We used logistic regression models to further examine this association. In the univariate analysis, factors including prenatal anxiety or depression, lower maternal and paternal educational level, nonvaginal delivery, nonurban residence, and lower household income were all significantly associated with increased risk of ASD. To assess the independent effect of prenatal anxiety or depression, we included all these variables along with age and sex in the multivariable logistic regression model. The results showed that maternal prenatal anxiety or depression remained a significant independent risk factor for ASD (adjusted OR 3.897, 95% CI 2.481-6.119). Although some other variables also retained significance, their effect sizes were smaller than that of prenatal anxiety or depression. These findings suggest that maternal prenatal anxiety or depression may independently increase the risk of ASD in children. This is consistent with previous cohort studies linking maternal anxiety or depression before birth to ASD or ASD-related traits [11,26,27] and with systematic reviews and meta-analyses reporting increased ASD risk among children of mothers with affective, depressive, or anxiety disorders [12,13]. Although the precise mechanisms remain to be clarified, both animal and human studies have demonstrated that maternal prenatal anxiety and depression may be associated with neurodevelopmental disorders [28]. As such, addressing and managing maternal prenatal anxiety and depression may be crucial for reducing the prevalence of neurodevelopmental disorders, including certain forms of ASD, in children.

GI symptoms are a common comorbidity in children with ASD [3]. In the present study, we found that 50.2% (236/470) of children with ASD had GI symptoms, which was significantly higher than the proportion of children with typical development with GI symptoms. Additionally, we compared the prevalence of GI symptoms in children with ASD whose mothers had and did not have prenatal anxiety and depression. The results showed that for children with ASD whose mothers had prenatal anxiety or depression, the prevalence of total GI symptoms was 62.3% (66/106), which was significantly higher than that of children with ASD whose mothers did not have prenatal anxiety and depression (170/364, 46.7%). Among the GI symptoms, the prevalence of constipation and abdominal pain was significantly higher in children with ASD whose mothers had prenatal anxiety or depression than in children with ASD whose mothers did not have prenatal anxiety and depression. This suggests a potential link between maternal anxiety and depression during pregnancy and GI symptoms in children with ASD. Furthermore, we used logistic regression models to analyze the relationship between maternal prenatal anxiety or depression and GI symptoms in children with ASD. The results suggested that maternal prenatal anxiety and depression might be risk factors for GI symptoms in children with ASD, with an increased risk of constipation, abdominal pain, and total GI symptoms. This trend remained consistent even after adjusting for potential confounding factors. It has been reported that there is a correlation between maternal depressive symptoms and infant gastroesophageal reflux disease and constipation [29]. However, to our knowledge, no other research has explored the link between maternal anxiety and depression during pregnancy and GI symptoms in children with ASD.

The mechanisms underlying the association between maternal prenatal anxiety and depression and ASD with GI symptoms in children are unknown. Depression and anxiety are highly comorbid with GI abnormalities as well as microbiome dysbiosis [30,31]. In the case of maternal depression during pregnancy, they can also have a significant impact on both the mother’s and the child’s gut microbiome [32]. A study conducted by Galley et al [14] also supports the idea that maternal prenatal anxiety and depression can affect the gut microbiome of children. Additionally, prenatal stress has been found to affect placental cytokines and neurotrophins [33]. Moreover, an animal study has suggested that maternal microbiome can modulate fetal neurodevelopment [34]. In addition, abnormalities in the development of the brain and ENS can contribute to the development of ASD and GI symptoms [35]. Thus, we speculate that maternal prenatal anxiety and depression may affect the development of children’s brains and GI tracts through abnormalities in maternal microbiota as well as abnormalities in placental function, or it may directly affect children’s gut microbiota and further influence brain development through the gut-brain axis, which may increase the risk of ASD and GI symptoms in children. However, the specific mechanisms need to be further explored in animal experiments.

### Limitations

This study has several limitations. First, it was a cross-sectional study, which does not allow for causal inferences to be made about the relationship between prenatal anxiety and depression and ASD with GI symptoms. In addition, some of the questionnaire data, including general demographic information and certain maternal mental health information, were collected retrospectively at enrollment when the children were aged 2 to 7 years and, thus, may be subject to retrospective recall bias. Second, there is no standardized and validated method for assessing GI symptoms in children with ASD. In this study, GI symptoms were judged based on the caregivers’ answers to questions based on the QPGS-IV, and children with ASD usually have communication problems, so there may be bias in evaluating GI symptoms. Third, although we adjusted for major confounding factors, our analysis may still have residual unmeasured confounding such as maternal fever, obesity, and diabetes, which were not included in the analysis and could influence the observed associations. Finally, as the ASD group was recruited from a specialized tertiary behavioral clinic while the typical development group was drawn from a routine well-child health clinic, there may be potential selection and referral biases due to differences in referral pathways and clinic attendance.

### Conclusions

Our study found that a higher proportion of children with ASD had mothers with prenatal anxiety and depression compared to children with typical development. Children with ASD whose mothers experienced anxiety or depression during pregnancy had significantly higher rates of GI symptoms than children with ASD whose mothers did not experience anxiety and depression during pregnancy. Our logistic regression models suggest that maternal prenatal anxiety and depression may increase the risk of ASD and GI symptoms in children. These findings highlight the importance of mental health during pregnancy and that even mild and moderate anxiety and depression may increase the risk of ASD and GI symptoms in children, so they need to be actively prevented and treated.

## Abbreviations

ASD: autism spectrum disorder
CARS: Childhood Autism Rating Scale
ENS: enteric nervous system
GI: gastrointestinal
OR: odds ratio
QPGS-IV: Questionnaire of Pediatric Gastrointestinal Symptoms–Rome IV
